# Hypogonadism and Intracranial Hypertension: A Case Report and Brief Review

**DOI:** 10.7759/cureus.23660

**Published:** 2022-03-30

**Authors:** Marc A Abboud, Trisha U Nguyen, Jordan M Smith, Kevin Campbell

**Affiliations:** 1 Urology, University of Florida, Gainesville, USA

**Keywords:** pituitary dysfunction, androgens, testosterone, hypogonadism, intracranial hypertension

## Abstract

Intracranial hypertension is rare and there are few cases in men in the literature that report an association between hypogonadism and intracranial hypertension. Herein, we review a diagnosis of hypergonadotropic hypogonadism in the setting of intracranial hypertension. The patient was a 40-year-old male with morbid obesity, hypertension, and prediabetes, with symptoms of hypogonadism, who on further workup was found to have intracranial hypertension. This case report serves to raise awareness of the association between idiopathic intracranial hypertension and hypogonadism in men.

## Introduction

Hypogonadism is defined by the American Urological Association as a total testosterone level below 300 ng/dL with associated subjective symptoms such as fatigue, decreased libido, and erectile dysfunction. Hypogonadism can be classified as primary or secondary. In primary hypogonadism, the testicles do not produce sufficient levels of testosterone. In response, there is decreased inhibitory feedback on the hypothalamic-pituitary axis. The pituitary gland increases the production of gonadotropins, including luteinizing hormone (LH) and follicle-stimulating hormone (FSH). This condition is known as hypergonadotropic hypogonadism. In secondary hypogonadism, the hypothalamus or pituitary gland demonstrates decreased gonadotropin release, which results in downstream decreased testicular testosterone.

In 2010, a cross-sectional study from the European Male Ageing Study found the prevalence of adult-onset hypogonadism to be approximately 13.8%, with 2.0% of the men diagnosed with primary hypogonadism [1]. Although various comorbidities have been associated with adult-onset hypogonadism, such as high body mass index, diabetes, asthma/chronic obstructive pulmonary disease, and prostate disease, there are few cases in the literature where hypogonadism has been associated with intracranial hypertension [1]. Herein, we review a diagnosis of hypergonadotropic hypogonadism in the setting of intracranial hypertension.

## Case presentation

**Figure 1:**
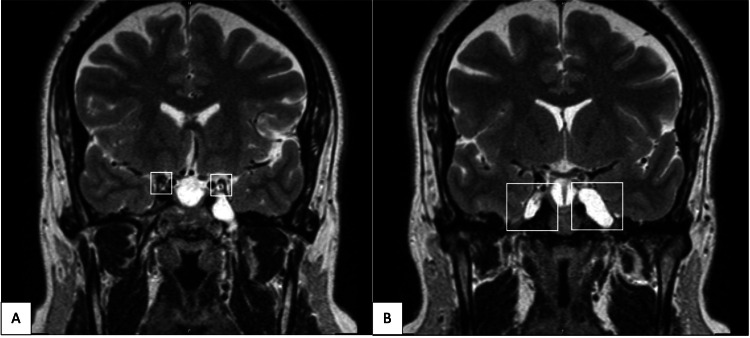
Coronal T2 brain MRI showing signs of intracranial hypertension. A: Dilated and fluid-filled oculomotor cisterns. B: Bilateral enlarged Meckel’s cave and bilateral tortuosity of optic nerves with increased CSF spaces.

A 40-year-old male with morbid obesity, hypertension, and prediabetes presented to his primary care clinic for evaluation of hypogonadism. He had initially been found to have low testosterone with a total testosterone level of 140 ng/dL when his levels were checked six years prior due to difficulty with weight loss. He deferred initiation of testosterone therapy (TTh) at that time and no further workup was pursued. His testosterone levels were rechecked five years later and again found to be decreased (total testosterone: 59 ng/dL; free testosterone: 8.3 pg/mL) with elevated gonadotropins (FSH: 14.4 mIU/mL; LH: 13.7 mIU/mL), consistent with hypergonadotropic or primary hypogonadism. He had regained approximately 100 pounds and wished to start TTh for weight loss and improved body composition. Scrotal ultrasound and sella MRI were performed as part of the workup prior to initiating therapy. The scrotal ultrasound revealed bilateral testicles in the inguinal canals with the left testes measuring 1.5 cc and the right testes measuring 2.5 cc. MRI demonstrated an enlarged sella with significant thinning in the pituitary parenchyma and bilateral tortuosity of the optic nerves concerning for idiopathic intracranial hypertension (IIH) (Figure 1). He was referred to urology and neurology for further evaluation.

At this stage, his symptoms of hypogonadism included weight gain, fatigue, increased adiposity, decreased muscle mass, mild erectile dysfunction, and gynecomastia. A karyotype was performed with unremarkable findings (46XY). A neurological workup did not reveal any symptoms significant for pseudotumor cerebri. He denied any pulsatile tinnitus, headache, vision changes, papilledema, changes in gait, bowel or bladder dysfunction, or issues with swallowing or chewing. He elected to forego a diagnostic lumbar puncture to measure intracranial pressure due to his lack of symptoms. Additional genetic testing was deferred by the patient.

Given his low blood testosterone level, elevated gonadotropins, and small testes, the patient was determined to have a component of primary testicular failure. Although his MRI had findings concerning for intracranial hypertension, his asymptomatic presentation and normal neurologic exam were reassuring. He elected to undergo TTh and will be followed closely to assess for side effects and efficacy.

## Discussion

IIH is rare in men. Only 9% of IIH patients are male, and men are twice as likely as women to suffer severe visual loss as a result of the disease but significantly less likely than women to report headache as their initial symptom. Additionally, men are significantly more likely to report visual changes as their first symptom compared to women [2]. This suggests that men with developing IIH are more likely to remain asymptomatic until the manifestation of visual changes.

There is a recognized association between IIH and hypogonadism in men. A case-control study of 24 IIH men and 48 matched controls found that men with IIH had 17 times the odds of hypogonadal symptoms based on the Androgen Deficiency in Aging Males (ADAM) questionnaire [3]. It appears that aberrations in androgen levels increase the risk of IIH rather than the converse. This is seen in both males and females: males with androgen deficiency and females with excess androgens [4,5]. IIH predominantly affects obese women of reproductive age, and androgens are known to play an important role in its pathogenesis [6,7]. Serum and CSF testosterone are significantly higher in women with IIH compared to women with polycystic ovarian syndrome (PCOS) or simple obesity. Additionally, androgen receptors found in the choroid plexus have been shown to modulate CSF output, suggesting a potential driver of disease and target for future therapy [7]. Several case reports of transgender patients receiving hormone therapy and a report regarding an advanced prostate cancer patient on long-term androgen deprivation therapy also support the idea that androgens play a key role in the pathogenesis of IIH [5,8-11].

Based on these findings, researchers have suggested a “pathophysiological window” of abnormal circulating androgen levels in humans that is above the normal range for females but below the normal range for males [4,8]. There are defined sexually dimorphic roles of androgens in metabolic diseases [10]. Men with androgen deficiency and women with androgen excess share a largely overlapping adverse metabolic phenotype. These levels of androgens are associated with preferential accumulation of visceral fat, insulin resistance, and nonalcoholic fatty liver disease [8,12]. Adipose tissue functions as an endocrine organ with secretions determined by its distribution [3,8]. Therefore, IIH may represent a neurological sequela of metabolic pathology secondary to abnormal circulating androgen levels, which are depressed for men and elevated for females [8]. Whether a direct manifestation of abnormal androgen levels [6,7] or sequela of intermediary metabolic pathology [8], there is a clear association between IIH and hypogonadism in men.

## Conclusions

As the presented patient was asymptomatic at the time of evaluation without clinically evident papilledema, he did not wish to undergo a lumbar puncture to rule out IIH. The diagnostic criteria for IIH may be met in the absence of papilledema if the following are present: normal neurological exam, normal CSF composition, elevated lumbar puncture opening pressure, and three of the following MRI findings including empty sella, flattening of the posterior aspect of the globe, distention of the perioptic subarachnoid space with or without a tortuous optic nerve, and transverse venous sinus stenosis. Based on this, the presented patient would need a lumbar puncture to rule in or out IIH.

This case report serves to raise awareness of the association between IIH and hypogonadism in men. Clinicians should not ignore MRI signs of elevated intracranial pressure and consider fundoscopic exam with referral to neuro-ophthalmology if appropriate, as men with IIH often present with visual changes as the initial symptom.
